# Influence of Portosystemic Shunt on Liver Regeneration after Hepatic Resection in Pigs

**DOI:** 10.1155/2009/835965

**Published:** 2009-09-24

**Authors:** R. Ladurner, M. Schenk, R. Margreiter, F. Offner, A. Königsrainer

**Affiliations:** ^1^Department of General, Visceral and Transplant Surgery, University Hospital Tübingen, Hoppe-Seyler-Str. 3, 72076 Tübingen, Germany; ^2^Department of General and Transplant Surgery, University Hospital Innsbruck, Anichstrasse 35, 6020 Innsbruck, Austria; ^3^Department of Pathology, Academic Teaching Hospital, Carinagasse 47, 6800 Feldkirch, Austria

## Abstract

*Objective*. The minimal amount of liver mass necessary for regeneration is still a matter of debate. The aim of the study was to analyze liver regeneration factors after extended resection with or without portosystemic shunt. *Methods*. An extended left hemihepatectomy was performed in 25 domestic pigs, in 15 cases after a portosystemic H-shunt. The expression of Ki-67, VEGF, TGF-*α*, FGF, and CK-7 was analyzed in paraffin-embedded tissue sections. *Results*. The volume of the remnant liver increased about 2.5-fold at the end of the first week after resection. With 19 cells/10 Glisson fields versus 4/10, Ki-67-expression was significantly higher in the H-shunt group. VEGF- and CK-7-expressions were significantly higher in the control group. No significant change was found in FGF-expression. The expression of TGF-*α* was higher, but not significantly, in the control group. *Conclusions*. The expression of Ki-67, and therefore hepatocyte regeneration, was increased in the shunt group. The expression of CK-7 on biliary epithelium and the expression of VEGF, however, were stronger in the control group.

## 1. Introduction

One of the fascinating aspects of the liver is its remarkable capacity to regenerate itself after injury. Even after major insult, such as surgical resection (the strongest stimulator of hepatic regeneration), the liver usually recovers sufficient function within two weeks [1, 2]. Liver mass is precisely regulated, and signals from the body have both positive and negative effects on liver volume increase until the proper size is reached in relation to body size. Instead of regeneration in the true sense, the residual tissue hypertrophies until the original mass has been restored, and once attained, liver regeneration halts abruptly [3]. As demonstrated by repeated liver resections in rats [4], and observed after re-resection in patients with recurrence of malignant liver tumours, the regenerative capacity of the liver is almost unlimited. However, the exact cascade of growth factors, as well as the correlation between liver cell proliferation and growth factor levels during liver regeneration in humans, remains unclear. Also, the regenerating liver, especially when only a critical liver mass is left, exhibits little reserve capacity. The remaining functional liver volume limits its own regeneration, as the liver must simultaneously exert its normal metabolic functions while undergoing proliferation. The question, therefore, concerning the minimal liver remnant has been the subject of much debate and countless studies [5]. One of the most discussed causes of liver failure after extended liver resection is the obligatory increase in portal blood flow through the remnant liver, resulting in centrolobular arterial hypoperfusion and sinusoidal damage. The effect of a portosystemic shunt on portal vein decompression has been considered an important factor in preventing progressive necrosis and ultimately fatal liver failure. But the role of portal blood flow after extended liver resection, as well as after transplantation of small-for-size grafts, is still not completely understood. On the one hand, an adequate portal blood supply is essential for hepatic regeneration [6, 7], in that portal blood delivers growth factors to hepatocytes from the gut, including TGF-*α* (transforming growth factor-alpha), epidermal growth factor, interleukin-6, and tumour necrosis factor, to name a few [8, 9]. On the other hand, previous studies have demonstrated that the damage of hepatic sinusoids resulted from transient portal hyperperfusion after small-for-size liver grafting or extended hepatectomy [10–12]. Several techniques such as splenic artery ligation [13, 14], portosystemic [12, 15], mesocaval shunting [16], and splenectomy [17] have been applied to attenuate portal hyperperfusion after reperfusion and liver resection.

The aims of the present study were to determine the influence of different factors such as Ki-67, VEGF (vascular endothelial growth factor), TGF-*α*, FGF (fibroblast growth factor), and CK-7 (cytokeratin 7) on small liver volume after extended hepatic resection with and without a portosystemic shunt in a large animal model.

## 2. Materials and Methods

The project and study design were approved by the Austrian Federal Animal Investigation Committee, and animals were treated in accordance with the National Institutes of Health guidelines. An extended left hemihepatectomy (approximately 75% of liver volume), as described previously [18], was performed in 25 healthy female German landrace pigs, 6–8 weeks old and weighing 25–35 kg. For blood and vessel procurement (infrarenal aorta) and also for volumetry of the liver, male sibling pigs were used as donors. Dividing the pigs among males and females was done in order to produce a uniform study group. The study animals were divided into two groups. The control group (*n* = 10 pigs) underwent extended liver resection alone. The shunt group (*n* = 15) received a side-to-side portosystemic H-shunt as an interposition graft (7 × 8 mm aortic allograft, diameter 7-8 mm) after partial clamping of the portal- and infrahepatic caval veins. The male pigs also underwent an extended left hemihepatectomy in order to measure (1) the liver volume being resected, (2) the remaining liver tissue after extended left hemihepatectomy, as well as (3) the total liver volume at the time of operation.

After surgery the animals were weaned from anaesthesia and extubated. In the shunt group, low-dose heparin (2 mg/kg/d) was administered. The animals were observed regularly under sedation with ketamine (5–10 mg/kg intramuscularly). Doppler ultrasound was performed to assess the patency of the shunt and the flow rates (mL/min) in the portal vein and in the right lateral artery. 

The liver volume being resected in the study was measured at the time of the operation and compared with the liver volume being resected in the male pigs. The liver volume of the remnant regeneration liver was measured at the end of the experiment. Since the total liver volume and the remnant liver volume at the time of surgery could not be measured exactly in pigs with extended liver resection, data from the male sibling pigs were used for comparison. 

After death or sacrifice, the animals were carefully examined to identify surgical complications. The remnant liver was excised for volumetric measurements and histological work up. The liver volumes were measured by water displacement and recorded in millilitres (mL). 

Tissue samples, obtained preoperatively and intraoperatively, by means of ultrasound-guided biopsy and postmortem examination, were fixed with formalin, embedded in paraffin and stained with hematoxylin-eosin. A total of 11 biopsies were examined in the control group (8 biopsies in the first, 2 in the second, and 1 biopsy in the third week after resection) and 24 biopsies in the shunt group (19 biopsies in the first, 3 in the second and 2 in the third week after resection and shunt-operation). Immunuhistochemistry, with regard to the mitogens Ki-67, VEGF, TGF-*α*, and FGF, as well as CK-7, was scored in the usual 4 categories, from 0 (no staining) to 3 (intensive staining). Histochemistry was analyzed blindly by two clinicallyexperienced observers, who were not involved in sampling or animal care. Both categorizations were included equally into the results.

## 3. Statistics

For continuous data the Wilcoxon rank sum using SPSS software was performed. A significance level of *P* < .05 was considered sufficient in all experimental groups. For histological grading the Likelihood Ratio was used. 

## 4. Results

Survival rates did not differ between the two groups. In each group 6 animals survived the first postoperative week, and two animals in each group were sacrificed in good condition on days 17 and 19, respectively. Three animals in the control group and two in the shunt group developed ascites at the end of the first week after surgery. Nevertheless, none of the animals in either group showed splenomegaly or congestion of the bowel, and no signs of congestion of the portal system appeared at histology. Due to these findings, it was concluded that 75% hepatectomy does not lead to portal hypertension in pigs.

The blood flow in the hepatic artery increased slightly, but not significantly in animals after extended liver resection without shunt: 49 ± 8 mL/min before resection and 79 ± 13 mL/min after resection. In the group with H-shunt the increase was also not significant: 50 ± 8 mL/min before resection and 55 ± 8 mL/min postoperatively. However, in animals with H-shunt the portal blood flow decreased significantly: 1203 ± 187 mL/min before resection and 370 ± 32 mL/min after resection, (*P* < .005). In the animals without shunt the portal blood flow was not latered: 717 ± 155 mL/min before resection and 534 ± 61 mL/min after resection, indicating no significant difference.

After resection of 75% of the liver volume, the regeneration rate of the right lateral segment and segment I—defined as remnant liver volume at followup/remnant liver volume after surgery—was not significantly different between the two groups (Table 1).

The remnant liver revealed a different time-dependent gross appearance during postmortem examination: until day 11, the livers showed severe steatosis with no significant difference occurring between the two groups. One pig in the shunt group at the time of death on day 11 and the remaining four animals in both groups, sacrificed on day 17 and 19, respectively, all revealed a liver of normal colour and consistency.

Histological examination indicated severe necrosis in three cases without, and in two cases with, portocaval shunt, whereas necrosis was mild in two pigs after resection alone and in three animals after resection and shunting. In terms of localization, slightly more necrosis was found in the centrolobular region in the resection group, whereas in the shunt group more necroses were found in the periportal area. One animal from each group, sacrificed on day 19, revealed completely normal histomorphological liver architecture. 

**Figure 1:**
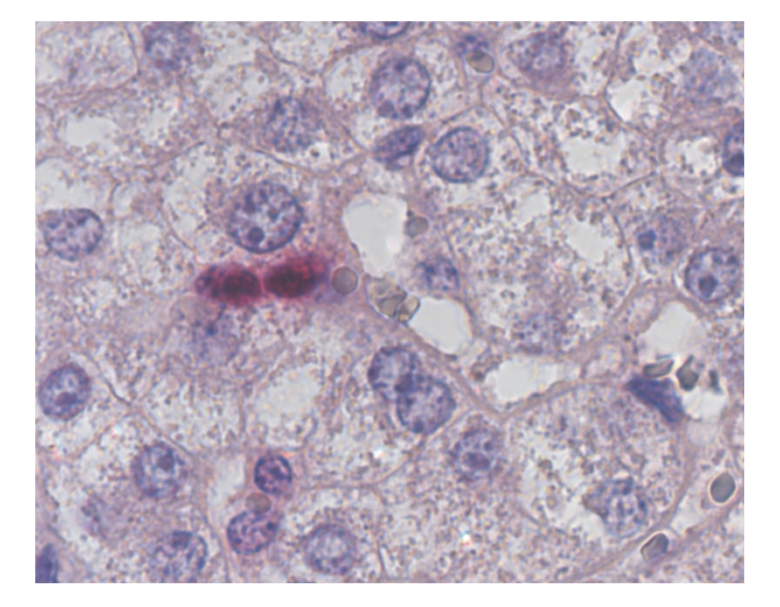
Hepatocyte undergoing cell division, stained by Ki-67, in the shunt group.

**Figure 2:**
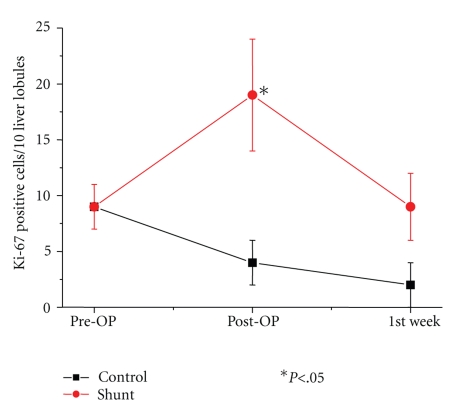
Mean number of Ki-67-positive hepatocyte nuclei in 10 liver lobules in the shunt (•) and in the control group (*▪*). With 19 cells-/-10 liver lobules versus 4-/-10, the Ki-67-expression approximately 4 hours after resection was significantly higher in the shunt group and remained at a higher level than in the control group. In the control group, the percentage of Ki-67 positive hepatocytes decreased slowly during the first week after resection. *P < .05.

Hepatocellular proliferation (Figure 1) was assessed approximately 4 hours after resection and during followup in the remnant liver by immunolabeling of Ki-67 nuclear antigen. It was quantified by counting the Ki-67-positive hepatocyte nuclei. With 19 cells-/-10 Glisson liver lobules versus 4-/-10, the Ki-67-expression immediately after resection was significantly higher in the shunt group, decreasing again in the first week after resection in the shunt group, but remaining clearly higher in the control group. In the control group, the percentage of Ki-67 positive hepatocytes decreased after resection (Figure 2).

With regard to TGF-*α* and FGF expression in liver tissue, no substantial difference was found between the two groups, although TGF-*α* production along the vessels was higher after resection as well as in the first postoperative week in the control group, rapidly decreasing thereafter. 

**Figure 3:**
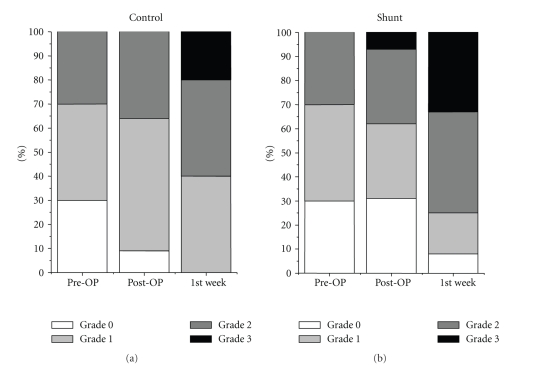
Total VEGF antigen expression in the liver parenchyma in the shunt and in the control group indicated a similar increase without a significant difference in both groups.

**Figure 4:**
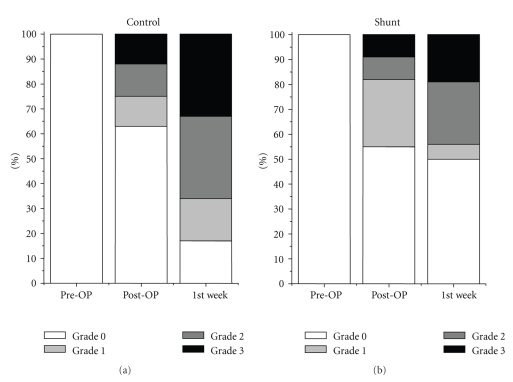
VEGF antigen expression around the central veins was significantly higher in the control group, not immediately after resection, but in the first postoperative week.

Total VEGF-antigen expression demonstrated a similar course occurring in both groups with an increase in the first week after surgery (Figure 3). The VEGF increase was significant in the first 7 days only around the central veins in the control group (Figure 4).

**Figure 5:**
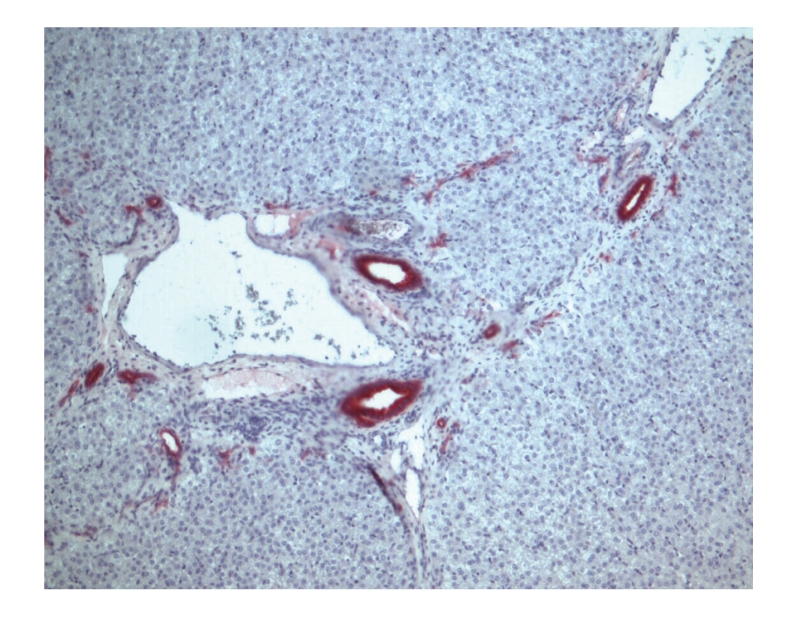
Bile ducts stained positive for CK-7 in the control group.

**Figure 6:**
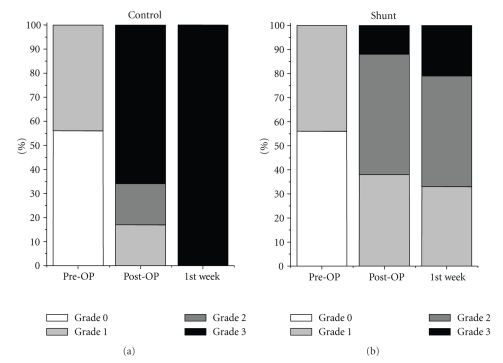
Extended hepatic resection alone was associated with a significant increase in CK-7 positive cells in the portal fields postoperatively and in the first postoperative week.

**Figure 7:**
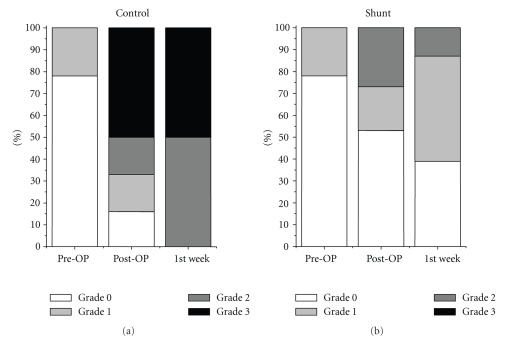
Extended hepatic resection alone was associated with a significant increase in CK-7 positive cells in the liver parenchyma postoperatively and in the first postoperative week.

Biliary epithelium was indicated by CK-7 expression within the liver parenchyma and the portal fields (Figure 5). Extended hepatic resection alone was associated with a significant increase in CK-7 positive cells in the bile ducts (Figure 6) as well as in the liver parenchyma (Figure 7) postoperatively and in the first postoperative week.

## 5. Discussion

Liver regeneration is a hyperplastic response and involves the proliferation of mature functioning cells composing the intact organ. Nevertheless, hepatic regeneration is a complicated process in which liver cells switch from a quiescent state to a proliferative state and re-enter the cell cycle. After extended resection or small-for-size transplantation, hepatic failure can closely be associated with impaired liver regeneration, caused by the damage of hepatic sinusoids resulting from excessive blood flow and transient portal hyperperfusion [10–12, 19]. The criticallysized graft with a calculated liver weight of less than 20% might be functionally adequate if we could protect it from portal flow-related injuries by maintaining intrasinusoidal hemodynamics within a “physiological” range that would not harm Kupffer cells or the endothelial lining cells [20]. Partial diversion of portal flow to systemic circulation through a portocaval shunt might therefore be a reasonable approach in reducing the risk of these injuries occurring. A portocaval interposition graft, or H-shunt, between portal vein and infrahepatic vena cava in our animal model was performed to reduce portal flow through the reduced vascular network of the liver remnant after resection and to avoid portal hypertension and irreversible injuries. The interposition graft permits a reduced hepatopedal flow and allows the passage of necessary regeneration factors from the intestinal tract, thereby promoting hepatic proliferation in the remnant liver. Finally, when liver regeneration is completed, the shunt can be easily occluded by interventional radiological embolization. Although portal perfusion in the shunt group was significantly reduced, extended hepatic resection with or without shunt in our animal study did not reveal significant differences between the two groups in terms of outcome, gross appearance, and histology, indicating comparable liver regeneration and leading us to the conclusion that portocaval shunting may only play a role within the first hours after extended liver resection. Our results, however, are in agreement with those of Rocheleau et al. [21]. Additionally, Man et al. [10] suggest that transient portal hypertension and excessive hepatic blood flow are found in the first 30 minutes after reperfusion of the small-for-size liver graft in a rat model. Also, although the portal hemodynamic changes are transient, the small-for-size graft injury reflected by biochemical and morphological changes is continuous and progressive, suggesting that irreversible sinusoidal damage occurred in the early phase after reperfusion.

Generally, immediately upon reduction of portal blood flow, the hepatic arterial buffer response is activated to maintain the constancy of hepatic blood flow, which is crucial for several homeostatic roles and finally for liver regeneration [22]. Nevertheless, the postoperative decrease of portal blood flow was significant only in the H-shunt group. The increase, though, of the hepatic artery blood flow in both groups was low and not significant. It still remains unclear from our study why the significantly reduced portal blood flow in the H-shunt group did not cause dilation and an important increase of the hepatic artery. However, the immediate postoperative hemodynamic imbalance with significant decrease in portal blood flow through the portosystemic shunt resulted in an excessive hepatocyte proliferation and an important increase in Ki-67-expression.

Hepatocytes, the main cellular components of the liver, are highly differentiated cells that rarely divide under physiological conditions; however, they do not lose their proliferative capacity or their ability to adapt to varying metabolic demands [23, 24]. These properties are quickly displayed when a reduction in hepatic mass occurs. Resection of liver tissue initiates the release of a cascade of growth factors that result in proliferation of all hepatic elements and the proliferation of the liver to its previous size. The intact liver is relatively unresponsive to exogenous growth factors, but rapid metabolic adaptations occurring almost immediately after partial hepatectomy render the remaining liver sensitive to extrahepatic and intrahepatic growth factors [25]. Nevertheless, the correlation between liver cell proliferation and growth factor levels during liver regeneration in humans remains unclear. 

Ki-67 is regarded as a marker of proliferation. It begins to be expressed in mid G1, through S and G2, and reaches its peak in the M phase. Hepatoctye proliferation was quantified by counting the Ki-67-positive hepatocyte nuclei per 10 liver lobules on tissue sections and revealed a highly significant difference, with more Ki-67-positive nuclei in the H-shunt group approximately 4 hours after resection. In the control group, the Ki-67-expression after resection was low and decreased slowly within the first postoperative week in contrast to the H-shunt group, leading us to the conclusion that the decrease of portal blood flow through a portosystemic shunt may have accelerated the beginning of hepatocyte proliferation. One week after liver resection, however, the difference between Ki-67-expression in the two groups was no longer significant. Although, in a liver resection model without shunt in mice, Makino et al. [26] showed the presence of only a very few Ki-67-positive hepatocytes, 3 to 10 hours after resection in 90% hepatectomized mice. In 70% hepatectomized mice, Ki-67-positive hepatocytes appeared after hepatectomy and proliferated thereafter. The authors therefore suggested that hepatic failure takes place in 90% hepatectomized mice because sufficient liver function needed to satisfy the metabolic demand is not provided, and cell death must be considered as failure of liver regeneration and the inability of the residual liver to maintain homeostasis. Furthermore, Corpechot et al. [27] postulated that Ki-67-positive hepatocytes are significantly increased in cirrhotic nodules, illustrating a low but permanently increased regenerative activity in cirrhotic livers; this activity is triggered by local hypoxia as a result of the impairment of sinusoidal permeability and perfusion. Nevertheless, in cirrhotic livers the capacity for regeneration after partial hepatectomy is significantly reduced. Although these models did not include a portocaval shunt, they demonstrate the high sensitivity of Ki-67-expression in hepatic proliferation and its susceptibility to hypoxia.

Other hypoxia-induced factors might be TGF-alpha and VEGF, as recently demonstrated [28]. VEGF promotes the proliferation of sinusoidal endothelial cells and the reconstruction of liver sinusoids [29, 30] and is up regulated within 24 hours after partial hepatectomy [31]. Whereas in our model, total VEGF-expression showed a slow increase within the first postoperative week in both groups, VEGF antigen expression around the central veins was significantly higher in the control group, not immediately after resection, but in the first postoperative week. The shunt, therefore, does not seem to influence significantly perivascular VEGF production in the periportal fields but may eventually influence the hypoxia-induced centrolobular VEGF-expression. Sturm et al. [32] reported a possible functional substitution of FGF by VEGF, demonstrating liver regeneration dynamics in FGF-deficient mice comparable with FGF-competent mice and underlining a functional synergism shown for FGF and VEGF in tumour angiogenesis. In our study, the course of FGF-expression in the parenchyma and vessels did not show any difference occurring between the two groups apart from a slight but not significant increase in FGF-expression in particles and tissue immediately after resection. 

In addition to VEGF, TGF-*α* seems also to be hypoxia-induced [28]. The shunt in our model again made no difference with regard to TGF-expression in the tissue. But in the vessels, TGF-*α*-expression in the first tissue samples after resection was high in the control group and increased more during the first postoperative week than in the group with H-shunt, where the postoperative level was already significantly lower and increased only slightly thereafter. Whether hypoxia was indeed the reason for this kind of TGF-expression remains unclear. The differences, however, were not significant.

Hepatocytes have a nearly unlimited capacity for self-renewal, a so-called “stem cell” property [33]. Such bipotential liver progenitor cells have been found in the developing liver of mice, rats, and humans [34] with the phenotype of both hepatocytes and biliary cells. Many studies indicate that bipotential liver progenitor cells may still exist in the adult rodent intrahepatic biliary tree [35, 36]. The precise anatomic localization of hepatic bipotential progenitors has not been well delineated in humans; however, a recent study points towards the canals of Hering as the likely niche [37] in the lobular phase. A subsequent breach of the basement membranes may be required for the local migration and/or intercalation of the progenitor cell-derived hepatocytes into pre-existing liver cell plates. Differentiation and migration of the progenitor cells towards bile ducts is also suggested by the observation of smaller CK-7 positive cells appearing amidst cuboidal epithelium of portal bile ductules and rarely amidst columnar cells of the larger interlobular bile ducts [38]. CK-7, however, is one of the markers used for identifying progenitor cells and biliary epithelium. In our study, CK-7-expression in the bile ducts and in the liver parenchyma was significantly higher in the control group immediately within the postoperative period and also in the first postoperative week, whereas in the shunt group CK-7-expression was observed in the bile ducts and very moderately in the liver parenchyma, though the increase was not significant. 

## 6. Conclusions

Understanding the physiological mechanisms of a liver volume which is too small is helpful in developing novel therapeutic and surgical strategies to avoid small-for-size syndrome in order to expand the indication for liver resection in hepatobiliary malignancies and to reduce or avoid small-for-size graft damage to the recipient as well as to prevent small-for-size syndrome in living donors in liver transplantation. In our large animal model, portocaval H-shunt after extended hepatic resection indicated an accelerated initial phase of regeneration by a significantly higher Ki-67-expression immediately within the postoperative period, a likely centrolobular, probably hypoxia-induced, elevated VEGF-expression in the control group, higher expression of TGF-*α* in the perivascular space in the control group, no significant difference in FGF production, and finally, a more pronounced influence of resection without shunt on biliary epithelium (CK-7-expression). Although a large number of growth factors can promote liver regeneration, the exact mechanism for this still remains unclear. Further studies are still necessary for understanding the impact of portal hyperperfusion, its advantages in liver regeneration, and its disadvantages in view of sinusoidal damage, in order to expand segmental liver transplantation and extended liver resection.

## Figures and Tables

**Table 1:** Regeneration of liver volume (liver volume at followup/remnant liver volume after surgery).

	With shunt	Without shunt
1st postoperative week	2.2 ± 0.1	3.0 ± 0.7
3rd postoperative week	6.5 ± 0.5	5.8 ± 0.8
